# Echocardiographic abnormalities in 124 severely malnourished adult anorexia nervosa patients: frequency and relationship with body composition and biological features

**DOI:** 10.1186/s40337-020-00343-y

**Published:** 2020-11-12

**Authors:** Mouna Hanachi, Annabel Pleple, Caroline Barry, Marika Dicembre, Emilie Latour, Maeva Duquesnoy, Jean-Claude Melchior, Abdallah Fayssoil

**Affiliations:** 1 Clinical Nutrition Unit, Raymond Poincaré Hospital (AP-HP), Garches, France; 2 Université de Versailles, Saint-Quentin-en-Yvelines, Montigny-le-Bretonneux, Versailles, France; 3 France INSERM, U1178 Paris, VI France; 4 Institut de Myologie, Groupe Hospitalier Pitié-Salpêtrière, APHP, Paris, France

**Keywords:** Heart abnormalities, Anorexia nervosa, Malnutrition, Echocardiography, Body composition

## Abstract

**Background:**

Anorexia Nervosa (AN) is a complex psychiatric disorder that can lead to specific somatic complications. Heart abnormalities are frequently reported, while their frequency and associated factors in severely malnourished AN patients remain poorly defined.

**Objectives:**

This study aimed to characterize echocardiographic abnormalities in severely malnourished AN patients and to assess associated clinical, biological and related body composition features.

**Methods:**

Between January 2013 and January 2015, all severely malnourished adult patients with AN (Mental Disorders, 4th Edn.-DSM IVr) were included in a monocentric study performed in in a highly specialized AN inpatient unit. Electrocardiogram (ECG) and echocardiography were used to assess both heart rhythm and function. All inpatients underwent a Doppler echocardiography procedure after undergoing combined blood volume adjustment, micronutrients deficiencies supplementation and electrolyte disorders correction. Right Ventricular (RV) and Left Ventricular (LV) systolic and diastolic functions were collected and compared to 29 healthy normal subjects in a control group.

**Results:**

One hundred and 24 patients (119 (96%) women, 5 (4%) men) with a mean age of 30.1 ± 11 years old and an average Body Mass Index (BMI) of 12 kg/m^2^ were included. Ninety patients (73%) had been diagnosed with AN Restrictive type (AN-R), 34 (27%) an AN Binge eating/Purging type (AN-BP). Eighteen patients (15%) disclosed an abnormal Left Ventricular Ejection Fraction (LVEF) (< 52% for male and < 54% for female). LVEF impairment was associated with AN-BP patients (*p* < 0.017) and hypertransaminasemia (AST and/or ALT ≥2 N) (*p* < 0.05). Left Ventricular mass (LV mass) and Left Ventricular End Diastolic Diameter (LVEDD) were significantly reduced in patients (*p* < 0.001, p < 0.001). Left and right ventricular tissue Doppler Imaging Velocities (TDI) peak were reduced in patients: Septal and Lateral LV Sm velocities peaks respectively 10 ± 2 cm/s (vs 14 ± 2 cm/s in controls, *p* < 0.001), 12 ± 3 cm/s (vs 16 ± 3 cm/s in controls, *p* < 0.001), basal RV Sm velocity peaks at 14 ± 3 cm/s (vs 19 ± 3 cm/s in controls, *p* < 0.001). Additionally, LV and RV diastolic velocity peaks were reduced: LV septal and lateral velocity peaks were respectively 13 ± 3 cm/s (vs 18 ± 2 cm/s *p* < 0.001), 12 ± 3 cm/s (vs 22 ± 4 cm/s, *p* < 0.001) and RV diastolic velocity peaks at 14 ± 3 cm/s (vs 21 ± 4 cm/s *p* < 0.001). LV diastolic velocity TDI peaks were significantly associated with hypertransaminasemia (*p* < 0.05) and tended to be associated with a low all body Fat-Free Mass Index (FFMI) (using Dual-energy X- ray Absorptiometry (DXA) (HOLOGICQDR 4500) (*p* = 0.056). Thirty-four patients (27%) had a pericardial effusion and were significantly associated with a decreased all body FFMI (*p* < 0.036).

**Conclusion:**

Heart abnormalities are frequent in malnourished patients with AN, particularly in AN-BP type. Both liver enzymes and body composition abnormalities tended to be associated with heart dysfunction (non-significant association). Prospective studies are needed to better characterize and describe the evolution of cardiac abnormalities during the refeeding period and subsequent weight restoration.

## Plain English summary

AN is a complex and severe eating disorder that affects 1–2.2% of young women. Its mortality rate is high, up to 12 times compared that of the general population. AN is defined as either an inability to maintain adequate dietary intakes associated with an intense fear of gaining weight or becoming fat, or a persistent behavior preventing weight gain and causing interference with body image.

Dietary restriction can cause undernutrition and serious medical complications such as heart abnormalities, which are frequent yet poorly defined.

We aimed to characterize echocardiographic abnormalities in severely malnourished patients suffering from AN and to assess associated clinical, biological and related body composition features. Results support that heart abnormalities are frequent in malnourished patients with AN (BMI < 12), particularly in the AN-BP type. Liver enzyme levels were associated with heart dysfunction. Body composition abnormalities tended to be associated with heart dysfunction. Our outcomes suggested that echocardiography may be performed in all severe malnourished AN-BP type patients on presentation, in order to diagnose and manage heart-related abnormalities, thereby reducing potential complications.

Prospective studies are needed to describe the evolution of these abnormalities after weight recovery.

## Introduction

Anorexia Nervosa (AN) currently affects 1% of young women [1]. With a mortality rate of 12 times that of the general population [2], AN is considered to be one of the most serious psychiatric eating disorders [1]. AN is defined as an inability to maintain adequate dietary intakes, to maintain normal weight for age, associated with an intense fear of gaining weight or becoming fat, or persistent behavior that interferes with weight gain and disturbances of the body image [3]. Two sub-types of AN are described, the pure restricting sub-type (AN-R), and the binge-eating/purging sub-type (AN-BP), combined with cycles of large meals that are followed by purging behaviors (vomiting and/or laxative abuse [3].

Serious medical complications have been reported, including electrolyte disorders, hematologic disorders [4], severe bone loss, fractures [5] and liver abnormalities [6]. Clinical and biological severity factors have been identified [7], including the reported complication of abnormalities affecting heart function [8]. The frequency of heart abnormalities varied between 0 and 76% depending on studies and may include myocardial dysfunction [9], arrhythmia, prolongation of the QT interval and sudden death [10]. The risk factors, pathophysiology and the cause for heart abnormalities observed in patients with AN remain under-investigated [11, 12].

In this study, we aimed to characterize echocardiographic abnormalities in extremely severe malnourished patients suffering from AN and to assess associated clinical, biological and related body composition features.

### Materials and methods

We conducted a retrospective study of all malnourished patients with AN (according to DSM IVr) [13] hospitalized for nutritional rehabilitation at Raymond Poincaré University Hospital, Garches; the largest specialized nutrition unit in France.

Data were collected for the time period between January 2013 and January 2015 and included patients with the following criteria: AN patients aged ≥16 years-old, BMI < 16 [14] with either a AN-R or AN-BP type. We excluded all patients with pre-existing heart or thromboembolic disease, inflammatory or septic disorders, hyper or hypothyroidism, and any pathology that might be associated with heart disease such as diabetes, infectious diseases and/or chronic inflammatory disease. ECG and echocardiography were used as tests of heart rhythm and function. Patients were compared to a control group of 29 healthy subjects, and were selected from the medical and paramedical staff in nutrition unit of Raymond Poincaré University Hospital (Garches, France). The control group underwent Doppler echocardiography performed by the same operator (AF) as for AN patients to reduce operator error.

### Patient management

During the first 48 h after admission, all patients underwent standard progressive enteral feeding, using standard polymeric enteral nutrition products, combined with initial intravenous supplementation of vitamins, phosphorus and trace elements. This therapeutic protocol was initially recommended by the NICE (National Institute for Health and Clinical Excellence) in the UK [7] and was subsequently confirmed by French National Health Authority (HAS) guidelines [15].

In cases where the BMI was below 12 and the patient exhibited hypophosphatemia at admission, patients were fasted, hydrated intravenously and given micronutrient supplements for the first 24 h. After the hydration period, enteral nutrition was initiated according HAS (France) and NICE (UK) recommendations, to avoid the occurrence of refeeding syndrome (RS) [16].

Enteral feeding was started gradually with the administration of 10 kcal / kilogram of body weight / day, followed by a gradual increase depending on clinical status and phosphatemia [17, 18]. No patients received parenteral nutrition. Oral nutrition was initiated gradually depending on the evolution of body weight under enteral refeeding by nasogastric tube. Clinical and biological data were collected weekly for 1 month.

### Studied parameters

For each patient the following clinical data were collected at admission: age, gender, AN types, height and weight. Blood biological parameters collected were as follows: Aspartate Transaminase (AST), Alanine Transaminase (ALT), Gamma Glutamyl- Transpeptidase (GGT), Alkaline Phosphatase (ALP), Prothrombin Rate (PR), and other biological parameters, such as: serum electrolytes, Urea, Creatinine, Phosphorus, Calcium, Magnesium, Calcifediol (25- OHD3), Thyroid-Stimulating Hormone (TSH) free Thyroxine (T4), Triiodothyronine (T3), Brain Natriuretic Peptide (BNP), Selenium, Thiamine, total Cholesterol and LDL (Low Density Lipoprotein), Triglycerides (TG), Albumin, Thransthyretin (TTR) and C-Reactive Protein (CRP).

Body composition Fat Mass (FM), Fat-Free Mass (FFM) and calculated Fat-Free Mass Index (FFMI) were studied using Dual-energy X-ray Absorptiometry (DXA) (HOLOGIC QDR 4500) measurements collected at admission.

Healthy normal weight (BMI between 18.5 and 25) volunteers were enrolled consecutively after a medical interview, among hospital’s medical and paramedical staff. Past and actual overweight (BMI > 25) volunteers were excluded. Controls did not undergo DXA or blood tests.

### Doppler echocardiography

After blood volume, electrolyte and/or micronutrient disorder correction, Doppler echocardiography were performed during the first week of hospitalization (median: 3 days) by an experienced AF, with a specialization in childhood and adult myopathic diseases, during the. Heart parameters were determined and interpreted according to the guidelines issued by the American Society of Echocardiography [19]. We recorded the following parameters: end-diastolic septal wall thickness (IVS), end-diastolic posterior wall (PW) thickness and end-diastolic LVdiameter (LVEDD), LV mass and left ventricular ejection fraction (LVEF). LVEF was considered normal if greater than 52% for male and 54% for female [20]. Tissue Doppler Imaging (TDI) was used to assess sub-clinical heart impairment, which allowed us to record, LV and RV systolic (Sm) and diastolic peak velocities (Ea) from the apical four chamber view. The tricuspid annular plane systolic displacement (TAPSE) recorded from the apical four chamber view using the M- mode cursor positioned at the free wall angle of the tricuspid valve annulus was used for the RV systolic function assessment [21].

RV systolic dysfunction was defined if TAPSE < 17 mm or peak RV systolic velocity (RV Sm) < 9.5 cm/s [20].

### Statistical analysis

Study results were analyzed using SPSS 11.0 (Chicago, Illinois, USA) software.

Student’s *t* test, chi-square test and Fisher’s test were used for univariate analysis. The risk factors identified using univariate analyses were tested using a multivariate logistic regression. A *p* value lower or equal to 0.05, was considered statistically significant.

## Results

### Patient characteristics at admission

One hundred and 27 inpatients were screened, 3 patients were excluded for concomitant comorbidities (one hypothyroidism, one diabetes and one pulmonary infection). One hundred and 24 were ultimately enrolled: 119 women (96%) and 5 men (4%). Ninety patients (73%) were suffering from AN-R type as defined by the DSM-IVr, while the other 34 (27%) presented AN with associated purgative behaviors (AN-BP). The mean age of patients was 30.1 ± 11 years old, with an average BMI of 12.3 ± 2.5 kg/m^2^ (BMI: 18.5–24.9 kg/ m^2^). Clinical and biological characteristics of the study population are summarized in Table 1. Twenty-nine healthy subjects, 28 women (97%) and 1 man (3%), aged 32 ± 10 years, represented the control group; their heart parameters were used for comparison to patients.

**Table 1:** Clinical and biological characteristics of patients at admission

	***N*** **= 124 patients**
Age (Y)	32 ± 2.8
Gender (M/F)	5 / 119
AN-R type	90 (73%)
AN-BP type	34 (27%)
Duration of AN since diagnostic (Y)	7.9 ± 5.7
BMI	12.3 ± 2,5
HR (bpm)	61.6 ± 17.2
SBP (mm Hg)	99.2 ± 12
DBP (mmHg)	65.6 ± 10.6
***Body composition (DEXA)***	
FFM (Kg)	***28.8*** ± 4.8
FM (%)	8.8 ± 3.8
FFMI (%)	10.8 ± 1.4
***Cardiac findings***	
QT interval (mm)	406 ± 54
QTc interval (mm)	385 ± 31
QT/QTc	1 ± 0.1
Pericardial effusion	34 (27%)
LVEF (%)	63 ± 10
LV mass (g)	80 ± 22
***Biological findings***	
Albumin (38–52 g/l)	36.9 ± 7.1
Transthyretin (0.21–0.45 g/l)	0.248 ± 0.102
CRP (< 5 mg/l)	6.5 ± 23.7
BNP (< 100 pg /l)	54 ± 70
Potassium (3.7–4.7 mmol/l)	3.8 ± 0.6
Calcium (2.12–2.52 mmol/l)	2.2 ± 0.1
Phosphorus (0.8–1.45 mmol/l)	1.1 ± 0.3
Magnesium (0.70–1.00 mmol/l)	0.8 ± 0,1
Patients with Hypertransaminasemia^a^	34 (27%)
AST (15–37 UI/l)	137.6 ± 425.8
ALT (12–78 UI/l)	170.1 ± 371.4
Total Cholesterol (C) (3.00–6.00 mmol/l)	4.2 ± 1.4
HDL-C (0.8–1.9 mmol/l)	1.8 ± 0.7
LDL-C (< 4.1 mmol/l)	2.1 ± 1.1
Total TG (< 1.7 mmol/l)	0.9 ± 0.7

Data are expressed as mean + SD or number (percentage)

*Y* Years, *M* Men, *F* Females, *AN-R* Anorexia Nervosa Restrictive type, *AN-BP* Anorexia Nervosa Binge Eating/Purging type, *BMI* Body Mass Index, *HR* Heart Rate, *SBD* Systolic Blood Pressure, *DBP* Diastolic Blood Pressure, *FFM* Free Fat Mass, *FM* Fat Mass, *FFMI* Free Fat Mass Index, *QTc* corrected QT interval, *LVEF* Left Ventricular ejection fraction, *LV* Left Ventricle, *CRP* C-Reactive Protein, *BNP* Brain Natriuretic Peptid, *AST* Aspartate Transaminase, *ALT* Alanine Transaminase, *HDL* High Density Lipoprotein, *LDL* Low Density Lipoprotein, *TG* triglycerides

^a^ = (AST and/or ALT> 2 N)

### Heart findings in patients and controls

A comparison of patients’ heart parameters and controls is presented in Table 2. At admission, 18 patients (15%) had a left ventricular systolic function < 52% (vs 0 subject in control group, *p* < 0.05). LV mass and LVEDD was reduced in patients (74.8 ± 23.4 g vs 110.77 ± 32.17 g, *p <* 0.001; 38 ± 5 mm vs. 44 ± 5 mm, *p* < 0.001respectively). Left and right ventricular TDI systolic velocity peaks were significantly reduced in patients: LV septal and lateral Sm velocity peaks respectively 10 ± 2 cm/s (vs 14 ± 2 cm/s *p* < 0.001), 12 ± 3 cm/s vs 16 ± 3 cm/s *p* < 0.001), basal RV Sm velocity peak 14 ± 3 cm/s (vs 19 ± 3 cm/s). In addition, LV and RV diastolic velocity peaks were both reduced: septal and lateral LV Ea velocity peaks respectively 13 ± 3 cm/s (vs 18 ± 2 cm/s *p* < 0.001), 12 ± 3 cm/s (vs 22 ± 4 cm/s, *p* < 0.001) and RV Ea diastolic velocity peak at 14 ± 3 cm/s (vs 21 ± 4 cm/s *p* < 0.001). TAPSE was significantly reduced in patients compared to controls (16.66 ± 3.95 mm vs. 22.8 ± 2.8 mm, *p* = 0.0002) (Table 2). Thirty-three patients (27%) presented mild to moderate pericardial effusion without heart impairment nor clinical signs of pericarditis. Two groups were identified according to their LVEF parameters: group A with an abnormal LVEF defined in males as < 52% and in females < 54%; group B with a normal LVEF > 52% in males and > 54% in females) [20] (Table 3).

**Table 2:** Echocardiographic data between patients and controls at admission

	Patients (***n*** = 124)	Controls (***n*** = 29)	***p***
BMI (kg/m^2^)	12 ± 2	21 ± 3	< 0.001
Age (Y)	32 ± 3	32 ± 10	NS
LVEF (%)	63 ± 10	66 ± 7	0.03
LV Mass (g)	74.8 ± 23.4	110.77 ± 32.17	< 0.001
LV Mass Indexed(g/m^2^)	43.20 ± 12.22	46.83 ± 10.77	0.07
LVEDD (mm)	38 ± 5	44 ± 5	< 0.001
IVS (mm)	6.92 + 1.39	7.73 ± 1.39	0.08
PW (mm)	7.3 ± 1.34	8.31 ± 1.38	0.07
TAPSE (mm)	16.66 ± 3.95	22.8 ± 2.8	< 0.001
Peak RV Sm velocity (cm/s)	14 ± 3	19 ± 3	< 0.001
Peak LV septal Sm velocity (cm/s)	10 ± 2	14 ± 2	< 0.001
Peak LV lateral Sm velocity (cm/s)	12 ± 3	16 ± 3	< 0.001
Peak RV Ea velocity (cm/s)	14 ± 3	21 ± 4	< 0.001
Peak LV septal Ea velocity (cm/s)	13 ± 3	18 ± 2	< 0.001
Peak LV lateral Ea velocity (cm/s)	12 ± 3	22 ± 4	< 0.001

Data are expressed as mean + SD or number (percentage)

*p p*-value, *NS* Not Significant, *BMI* Body Mass Index, *Y* Years, *LVEF* Left Ventricular ejection fraction, *LV mass* Left Ventricular Mass, *LV Mass Indexed* Left Ventricular Mass Indexed, *LVEDD* Left Ventricular End Diastolic Diameter, *IVS* End Diastolic Left Ventricular Septal Thickness, *PW* End-Diastolic Posterior Wall Tickness, *TAPSE* Tricuspid Annular Planeystolic Displacement, *RV* Right Ventricle, *LV* Left Ventricle, *Sm* Systolic Myocardial Velocity recorded withTissue Doppler Imaging; Ea: Early Diastolic Myocardial Velocity recorded with Tissue Doppler Imaging

**Table 3:** Comparison patients with and without heart failure (defined by LVEF Fraction < 52% for male and 54% for female)

	Patients heart failure (***n*** = 18)	Patients without heart failure(***n*** = 107)	***p***
Age (Y)	28 ± 5	32 ± 3	NS
BMI (kg/m^2^)	11.4 ± 1.6	12.3 ± 2.5	NS
FFMI (kg)/weight^2^)	10.49 ± 1.0	10.18 ± 1.2	NS
FM (%)	9 ± 2	8.8 ± 4	NS
AN types:			
AN-R	7 (50%)	84 (76%)	NS
AN-BP	7 (50%)	27 (24%)	0.16
Years of evolution of AN since AN diagnosis	11 ± 5	9 ± 7	NS
Selenium (mmol/l)	1 ± 0.1	1.4 ± 0.1	NS
Potassium (mmol/l)	4 ± 0.6	3.8 ± 0,6	NS
Calcium (mmol/l)	2 ± 0.2	2.2 ± 0.1	NS
Magnesium (mmol/l)	1 ± 0.2	0.8 ± 0.1	NS
BNP (pg/l)	60 ± 63	54 ± 63	NS
Albumin (g/l)	34 ± 9	37 ± 7	NS
Transthyretin (mg/l)	0.218 ± 0.096	0.248 ± 0.102	NS
CRP (mg/l)	5 ± 8	7 ± 23	NS
Hypertransaminasemia (% patients)	71%	43%	< 0.05
AST (UI)	290 ± 577.4	137.6 ± 425.8	
ALT (UI)	280 ± 439.7	170.1 ± 371.4	
HR (bpm)	70 ± 25	61 ± 17	NS
QT (ms)	402 ± 59	405 ± 54	NS
QTc (ms)	397 ± 30	388 ± 33	NS
QT/QTc	1.0 ± 0.1	1 ± 0.2	NS

Data are expressed as mean + SD or number (percentage)

*p* p-value, *NS* Not Significant, *Y* Years, *BMI* Body Mass Index, *FFMI* Free Fat Mass Indexed, *FM* Fat Mass, *AN* Anorexia Nervosa, *AN-R* Anorexia Nervosa Restrictive type, *AN-BP* Anorexia Nervosa Binge Eating/Purging type, *BNP* Brain Natriuretic Peptide, *CRP* C-Reactive Protein, *AST* Aspartate Transaminase, *ALT* Alanine Transaminase, *HR* Heart Rate, *QTc* corrected QT interval

### Factors associated with heart function abnormalities

Using univariate analysis, patients with AN-BP had a higher incidence of LV dysfunction compared to patients with AN-R (*p* < 0.017). Patients with LV systolic dysfunction frequently displayed hypertransaminasemia in comparison to patients with normal systolic function (*p* < 0.05). Using multivariate analysis, hypertransaminasemia and AN-BP were independently associated with LVEF alteration (*p* = 0.016; *p* < 0.05). LV TDI diastolic peak velocities (Ea septal peak and Ea lateral peak) were significantly associated with hypertransaminasemia (*p* = 0.05); there was an additional trend toward an association with FFMI, (*p* = 0.056). TDI Right ventricular diastolic velocity peaks ^(^RV Ea peak) were above the lower limit of normal in all AN patients. Considering an elevated BNP value (> 100 pg/ml) as a parameter indicative of altered left ventricular function, abnormal BNP values (> 100 pg/l) were significantly associated with older age (38 vs. 29 years old, *p* < 0.05) and with hypertransaminasemia (*p* = 0.001). A decreased FM was significantly associated with pericardial effusion (FM 2.4 kg in patients with pericardial effusion, vs. 3.1 kg in patients without pericardial effusion; *p* = 0.036).

## Discussion

In this study, we found that all severely undernourished AN patients had sub-clinical myocardial impairments and 15% of them displayed LV systolic dysfunction.

Cardiac abnormalities were associated with AN-BP type and hypertransaminasemia.

Free Fat Mass tends to be associated with LV diastolic function. Pericardial effusion was frequent and its presence was associated with a low FM.

Our results highlight the relationship between both heart and liver functions.

Characterization of heart abnormalities may lead to a better understanding for anticipating morbi-mortality in the future; though to our knowledge, no studies so far have focused on heart function (right and left, systolic and diastolic function) in AN patients. As for other disease settings, myocardial function may be altered in the context of metabolic disorders, such as acid-base disorders, hyperkalemia, hypomagnesaemia and hypocalcaemia [22]. These alterations, frequent in the context of AN, may also be associated with arrhythmia, and even with sudden death [23] though mitigating metabolic disorders can restore heart function [9]. This type of heart disorder was not studied and was therefore not covered by the present report. As for heart dysfunctions specific to AN, eight studies were published between 1994 and 2017, including seven comparing patients versus a control group. The number of patients included in these studies ranged from 11 to 91 [24, 25], mainly teenagers or very young adults with an average BMI 15 kg/m^2^. Overall four studies provided data on LV systolic function especially in very young patients, with outcomes within the normal ranges in all cases. The other studies reported a difference in structural parameters, but only two studies [25, 26] discussed subclinical systolic alterations, observed in 23 patients. Authors included structural parameters and LVEF in AN patients when echocardiographic assessment was performed. *Kastner* et al [11] carried out a large study of 173 children and adolescent patients with AN (age ranged from 12 to 17 years) and reported a frequency of pericardial effusion at 34.7% and reduced LVEDD in observed patients. Our study is one of the few that have addressed adult patients (mean age: 32 years old), with the largest number of patients having severe undernutrition (average BMI at 12 kg/m^2^). The Doppler-echocardiographic analysis was the most thorough compared with all others studies, focusing not only on the LVEF but also on subclinical systolic and diastolic function assessment. Our study was thus able to identify risk factors for subclinical heart alterations (FFMI, hypertransaminasemia, AN-BP type and elevated BNP). One of the major risk factors identified in this study was AN-BP type which is characterized by vomiting and laxative misuse potentially leading to hypokalemia, hypomagnesaemia and metabolic alkalosis. However these electrolyte disorders were not observed in our study because they were corrected intravenously in the early days of hospitalization, before refeeding. One can suppose that chronic metabolic disorders may alter myocardial fibers and that the damage remains even after micronutrient deficiency correction and subsequent recovery. This hypothesis was supported by both Romano et al. and Oflaz et al. [24, 26]. It would be interesting to perform heart evaluation a few weeks after undernutrition recovery to confirm or reject this hypothesis. Finally, particular focus should be on the heart function of AN-BP patients. *Rautou* et al reported on the liver biopsy of 12 malnourished AN patients with acute liver insufficiency, hepatocyte autophagia, liver hypoxia and decreased portal pressure. These microscopic abnormalities were associated with heart output. This low heart output appeared to be related to heart muscle atrophy and heart arrhythmia, which had been enhanced by electrolyte disorders, leading to hypokalemia and hypocalcaemia, and inducing QT prolongation and impairment of the autonomic nervous system [27]. Hypertransaminasemia was significantly associated with subclinical diastolic function impairment. In addition, a low FFMI tended to be associated with subclinical impairment of heart diastolic function. Hypertransaminasemia is usually frequent when the patients’ BMI is low [6]; this was our observation in this present study, and is corroborated by a research study carried out within our working group [6]. as well as in other literature [12].

Our study found a relationship between the level of severe undernutrition and heart alterations, highlighting the potential specific association between muscle mass and hepatic and cardiac impairment as a consequence of severe undernutrition.

The results of our study suggest that cardiac assessment for AN patients with hypertransaminasemia may be warranted prior to refeeding (or early in the refeeding algorithm) in order to prevent complications such as heart failure.

## Conclusion

Heart abnormalities are frequent in severely malnourished AN patients. LV function can be altered especially in AN-BP patients. Subclinical heart abnormalities are also frequent and tend to be associated to body composition abnormalities. Prospective studies are needed to determine and to describe the evolution of these heart abnormalities during refeeding and after weight restoration.

## Data Availability

The datasets used during the current study are available from the corresponding author on reasonable request.

## Abbreviations

AN: Anorexia Nervosa
DSM: Diagnostic and Statistical Manual of Mental Disorders
RV: Right Ventricular
LV: Left Ventricular
BMI: Body Mass Index
AN-R: Restrictive type
AN-BP: Binge eating/purging type
LVEF: Left Ventricular Ejection Fraction
AST: Aspartate Transaminase
ALT: Alanine Transaminase
LV mass: Left Ventricular mass
LVEDD: Left Ventricular end-Diastolic Diameter
TDI: Tissue Doppler Imaging
NICE: National Institute for Health and Clinical Excellence
HAS: French National Health Authority; RS: Refeeding Syndrome
GGT: Gamma Glutamyl-Transpeptidase
ALP: Alkaline Phosphatase
PR: Prothrombin Rate
25-OHD3: Calcitriol
TSH: Thyroid-Stimulating Hormone
T4: Thyroxine
T3: Triiodothyronin
BNP: Brain Natriuretic Peptide
CRP: C-Reactive Protein
FM: Fat Mass
FFM: Fat Free Mass
DXA: Dual-Energy X- Ray Absorptiometry
IVS: Septal Wall Thickness
PW: Posterior Wall Thickness
TAPSE: Tricuspid Annular Plane Systolic Displacement
